# Whole Genome Characterization and Genetic Evolution Analysis of a New Ostrich Parvovirus

**DOI:** 10.3390/v12030334

**Published:** 2020-03-19

**Authors:** Kunpeng Yuan, Dongdong Wang, Qingdong Luan, Ju Sun, Qianwen Gao, Zhiyao Jiang, Shouchun Wang, Yijun Han, Xueting Qu, Yueying Cui, Shimei Qiu, Youxia Di, Xiaoyi Wang, Shige Song, Peiheng Wang, Shilong Xia, Yongle Yu, Weiquan Liu, Yanbo Yin

**Affiliations:** 1 College of Veterinary Medicine, Qingdao Agricultural University, Qingdao 266019, China; 18306390700@163.com (K.Y.); lqddmj@126.com (Q.L.); gqwdmj2020@163.com (Q.G.); jzydmj@sina.com (Z.J.); wangshouchun2011@sina.com (S.W.); hyjdmj@souhu.com (Y.H.); qxtdmj@aliyun.com (X.Q.); cyydmj@tom.com (Y.C.); qsmdmj@tom.com (S.Q.); 2 Qingdao Bolong Experimental Animal Co., Ltd., Qingdao 266225, China; wdddmj@126.com (D.W.); sjdmja@126.com (J.S.); 3 China Ostrich Farming and Development Association, Beijing 100026, China; diyouxiayouxiang@163.com (Y.D.); wangxiaoyiyx2020@163.com (X.W.); ssgyouxiang2020@163.com (S.S.); wph2020@163.com (P.W.); xslyx2020@163.com (S.X.); 4 College of Biological Sciences, China Agricultural University, Beijing 100193, China; yylyx2020@163.com

**Keywords:** ostrich, leg paralysis, parvovirus, phylogenetic analysis, whole genome amplification

## Abstract

Ostrich diseases characterized by paralysis have been breaking out in broad areas of China since 2015, causing major damage to the ostrich breeding industry in China. This report describes a parvovirus detected in ostriches from four different regions. The entire genomes of four parvovirus strains were sequenced following amplification by PCR, and we conducted comprehensive analysis of the ostrich parvovirus genome. Results showed that the length genomes of the parvovirus contained two open reading frames. Ostrich parvovirus (OsPV) is a branch of goose parvovirus (GPV). Genetic distance analysis revealed a close relationship between the parvovirus and goose parvovirus strains from China, with the closest being the 2016 goose parvovirus RC16 strain from Chongqing. This is the first report of a parvovirus in ostriches. However, whether OsPV is the pathogen of ostrich paralysis remains uncertain. This study contributes new information about the evolution and epidemiology of parvovirus in China, which provides a new way for the study of paralysis in ostriches.

## 1. Introduction

Parvoviruses are small, non-enveloped, linear single-stranded DNA viruses [1]. Parvoviruses may have appeared millions of years ago, infecting invertebrates and vertebrates [2]. All parvoviruses that infect vertebrates belong to the parvovirinae subfamily [3]. Parvoviruses are widespread in birds; parvoviruses that cause harm to the health of avians mainly include goose parvovirus (GPV), Muscovy duck parvovirus (MDPV), and chicken and turkey parvoviruses [2]. Recent studies have shown that the diversity of known parvovirus species has greatly expanded and the host range of parvovirus may include the entire animal kingdom [4].

In recent years, an outbreak of disease has occurred in farmed ostriches aged from 1 to 4 months, with paralysis as the main clinical manifestation, and an incidence rate ranging from 30% to 80%. Sick ostriches gradually become thin and weak, with the disease lasting for about one month from onset to death. However, no visceral lesions have been found by gross examination in the heart, liver, lung, kidney, or spleen of sick ostriches. Small bleeding points in leg muscles, accompanied by increased joint fluids, have been observed in a few animals, but no visible lesions have been detected in most infected ostriches. Antibacterial drugs and antiviral drugs, such as ribavirin, have been ineffective in the treatment of the disease. While paralysis of nestlings has caused extensive loss within the ostrich farming industry in China, no effective solution has been reported so far.

Here, we describe a hitherto unknown ostrich parvovirus (OsPV), detected in paralyzed ostrich nestlings from the Beijing municipality and Hebei, Shanxi, and Yunnan provinces. Whole genomic sequencing and phylogenetic analyses have been carried out on strains, from each region, and their molecular evolutionary relationship with goose parvovirus (GPV) and Muscovy duck parvovirus (MDPV) established.

## 2. Materials and Methods

### 2.1. Sample Collection and PCR Detection

Sources of pathological materials were paralyzed ostrich nestlings from different farms in Beijing, Yunnan, Shanxi, and Hebei (Table 1). Livers, spleens, hearts, and brain tissues were collected from dead animals, and samples were homogenized in PBS, followed by freezing and thawing (3 times) and centrifugation for the collection of supernatants. DNA was extracted using a MiniBEST Viral RNA/DNA Extraction Kit Ver.5.0 (Takara, Beijing, China), according to the manufacturer’s instructions. The DNA of the samples was preserved at −20 °C. PCR was carried out according to the procedure of Tatár-Kis et al. [5], and the primers were according to the method of Zadori Z et al. [6]. All of the positive products were sequenced by Ruibiotech, Qingdao, China. Sampling was carried out by a veterinarian, who took different samples as part of his routine work and under the permission of the farm owner. For this reason, sampling did not require the approval of the Ethics Committee.

### 2.2. Whole Genome Amplification of OsPV

The primers were designed as described by Li et al. [7] to amplify the complete genomes of the OsPV strains. The genomes were amplified using PrimeStar HS DNA polymerase (TaKaRa, Beijng, China). PCR conditions were as follows: initial denaturation at 95 °C for 5 min, followed by 33 cycles of 95 °C for 45 s, 55 °C for 45 s, 72 °C for 60 s, and termination for 10 min at 72 °C. Amplified DNA fragments were visualized after electrophoresis on a 1% agarose gel (Tsingke, Beijng, China). PCR products were purified using a PCR purification kit (Cwbiotech, Beijng, China)., cloned into the pMD18-T vector (Takara, Beijing, China), and were sequenced by Ruibiotech, Qingdao.

### 2.3. Sequence Alignment and Phylogenetic Analysis

Sequence alignment and homology comparison based on nucleotide sequences between the four OsPVs obtained in this study, and other parvovirus sequences published in GenBank (Table S1), were conducted using Clustal W within the MegAlign program (DNASTAR Inc., Madison, WI, USA). and the MEGA program was used for pairwise distance analysis Proteins with motifs and domains were sought using profile hidden Markov models deployed by the HMMER server (https://www.ebi.ac.uk/Tools/hmmer/). RDP version 4.0 software was used for analyzing the recombination in the four OsPVs, to understand the relationship of OsPVs with GPV, MDPV. Phylogenetic trees based on replication (Rep) protein and capsid (Cap) protein sequences was constructed by using the maximum likelihood method with a Poisson model, based on 1000 bootstrap duplicates. Bootstrap values > 70% were considered to be significant. The nucleotides of OsPVs and classical avian parvovirus were compared by MegAlign, and the homology between OsPV and other avian parvovirus was analyzed.

## 3. Results

Parvoviral DNA was detected in a number of tissue samples (Table 1). Four positive samples, from Yunnan (OsPV-YN, Genbank Accession No.MK281604), Shanxi (OsPV-YQ, Genbank Accession No. MK281605), Hebei (OsPV-SJZ, Genbank Accession No. MK281603), and Beijing (OsPV-BJ, Genbank Accession No. MK281602) were sequenced. Results showed that the OsPV genome contained two major open reading frames (ORFs), which were similar to goose and Muscovy duck parvoviruses. The genome contains two open reading frames (ORFs), which can be divided into left ORF and right ORF. The left ORF encodes two replication (Rep) proteins, Rep1 and Rep2 [7]. The right ORF encodes three capsid (Cap) proteins named VP1, VP2, and VP3 [6]. The length genomes ranged from 5041–5103 nt containing 416–446 nt inverted terminal repeats (ITRs). The left ORF contained 1884 nt that encoding the Rep protein with 628 amino acids, while the right ORF of 2199 nt coded for the Cap protein with 733 amino acids. The nucleotide homology of the four OsPV genomes ranged from 99.0–99.4%. The nucleotide homology of the encoded Cap protein was 98.8–99.4% while that of the encoded Rep protein was 99.0–99.7%. The highest homology, 99.4%, was between the Shanxi and Hebei strains.

### 3.1. Gene Homology Comparisons with Other Avian Parvoviruses

Table 2 provides a gene homology comparison of OsPV, GPV, NGPV (parvovirus isolated from duck tongue disease), and MDPV. The OsPVs showed higher homology with GPV and NGPV—especially the GPV RC16 strain—than with MDPV.

### 3.2. Sequence Analysis among OsPV, GPV, and NGPV

**Figure 1:**
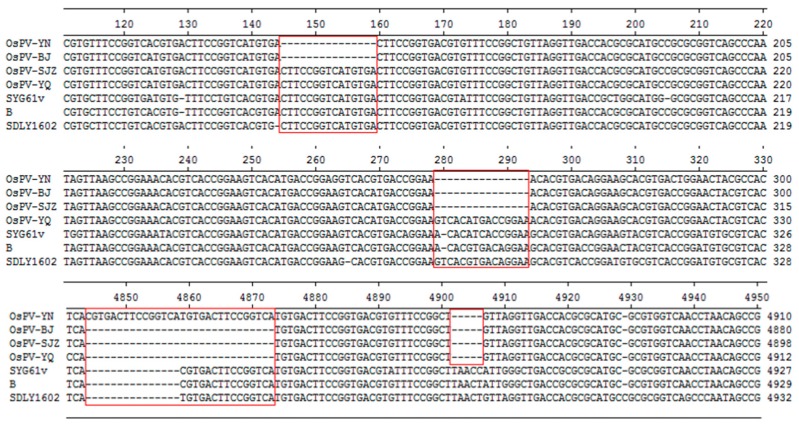
Sequence alignments of the inverted terminal repeats (ITRs) of the OsPVs, GPVs (goose parvovirus strains SYG61v and B) and NGPV (novel goose parvovirus related virus strain SDLY1602).

OsPV-YN and OsPV-BJ have 15 nt deletion at 145 nt, OsPV-YN, OsPV-BJ, and OsPV-SJZ have 15nt deletion at 279 nt, OsPV-YN has 15nt insertion at 4844. OsPV-BJ, OsPV-SJZ, and OsPV-YQ have 15nt deletion at 4859 nt (Figure 1).

### 3.3. Phylogenetic Analysis

**Figure 2:**
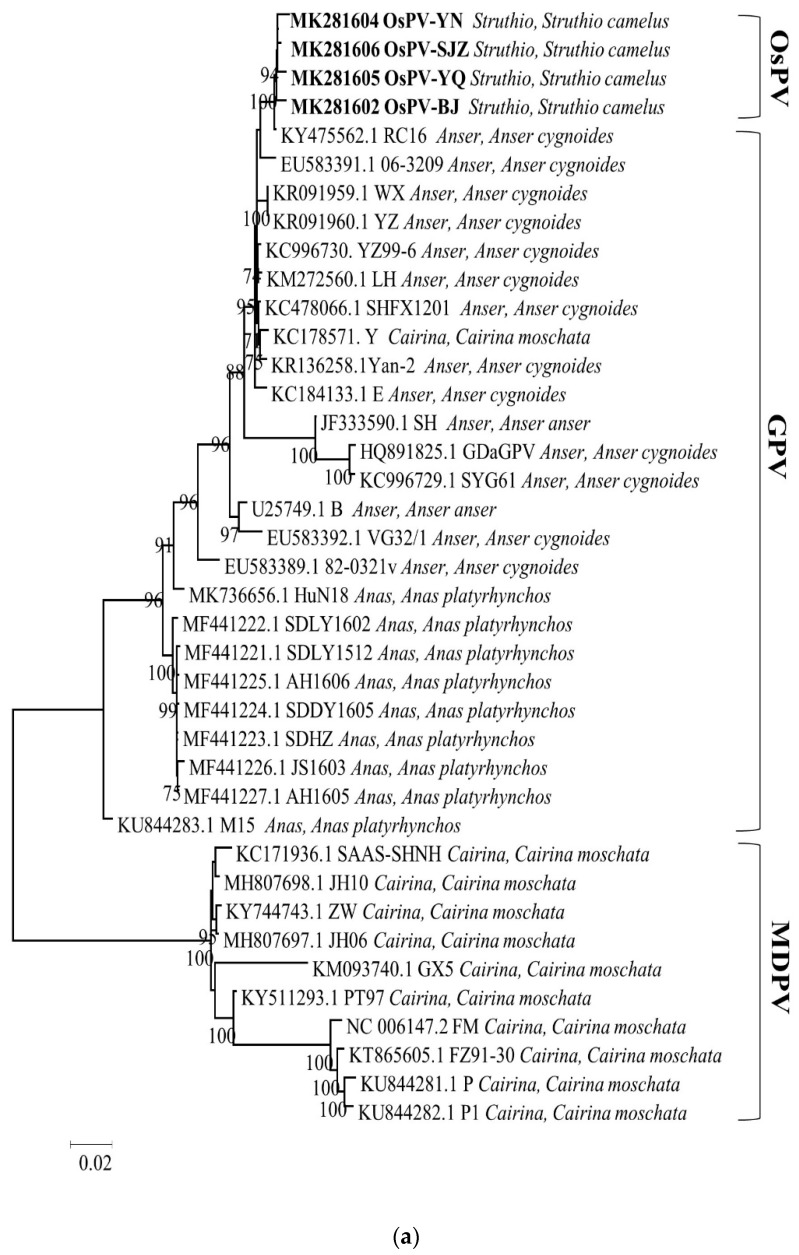

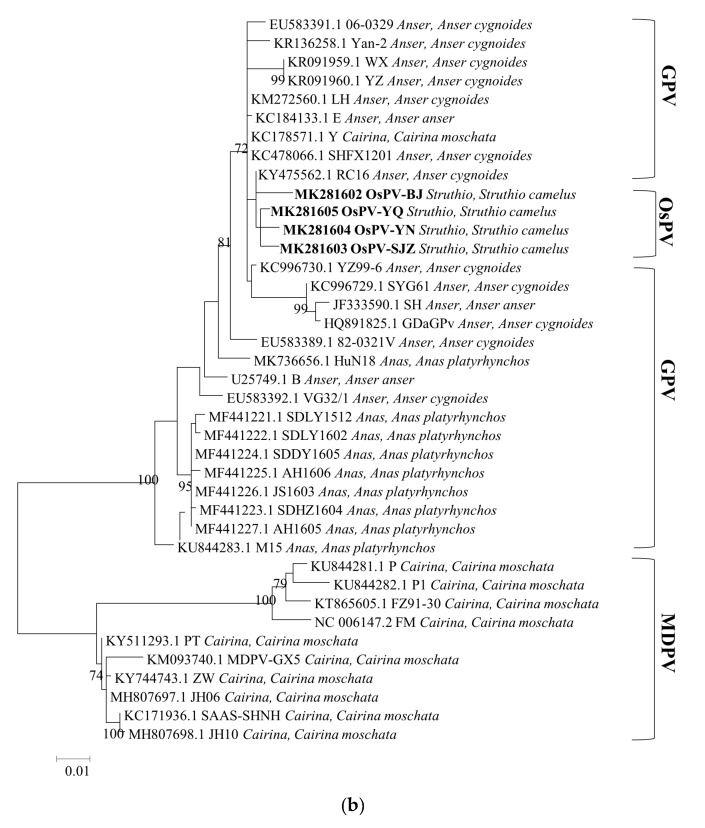

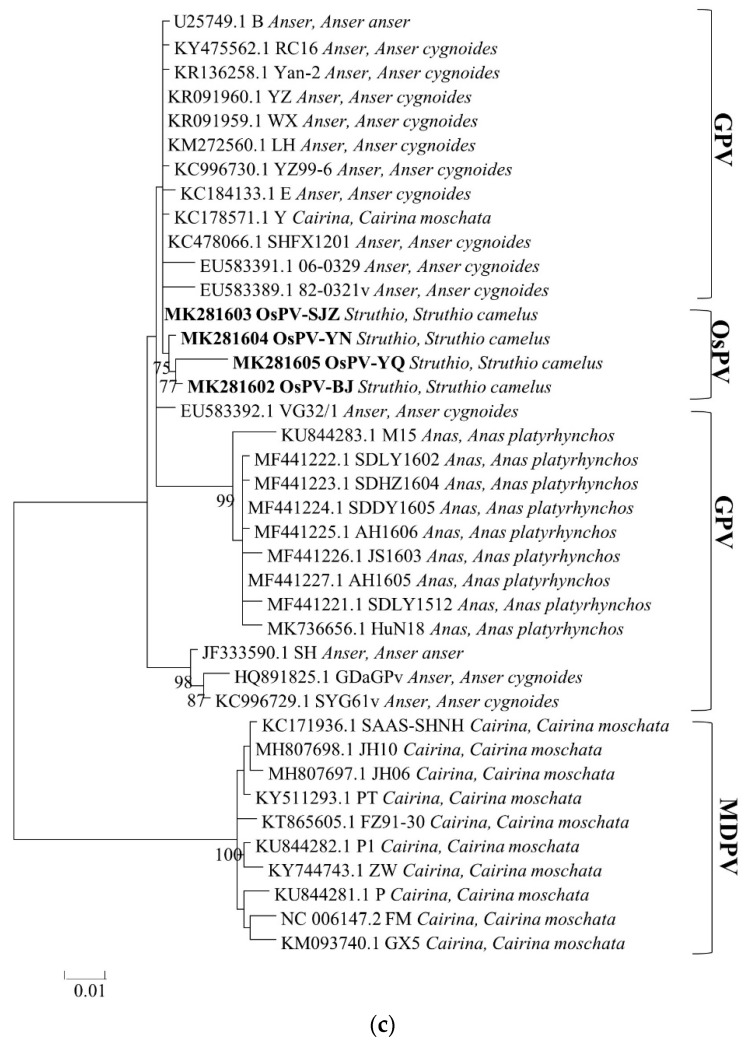
Phylogenetic trees based on complete genomic sequence (**a**), capsid proteins (**b**), replication proteins (**c**) were constructed by using the maximum likelihood method with a Poisson model, based on 1000 bootstrap duplicates. Bootstrap values > 70% were considered to be significant.

Phylogenetic analysis of the whole genome, the sequences of Rep protein and Cap protein. (Figure 2) revealed a close genetic relationship between the four OsPVs and GPV RC16 strains (goose parvoviruses isolated in Chongqing in 2016), forming a separate branch. The phylogenetic and alignment analysis showed that OsPV is a branch of GPV. There was a greater genetic distance between the OsPVs and SYG61, and an even greater one between the OsPVs and NGPV.

## 4. Discussion

In our study, OsPVs were detected in paralyzed nestlings from four different areas, which indicates that these viruses are widely distributed in ostrich farms in China. Use of yolk antibody against gosling plague to treat paralyzed ostrich nestlings in a number of farms was ineffective. In the absence of experimental animal infection, whether OsPV is the pathogen of ostrich paralysis remains uncertain. Phylogenetic analysis showed that OsPV is a branch of GPV, and OsPV strains were most closely related to the GPV RC16 strain isolated from Chongqing, China, in 2016. The replication protein of OsPV-SJZ and adeno-associated virus (AAV2) have the least E-value (3e-176), the capsid protein of OsPV-YN and AAV have the least E-value (1.4e-258), according to the search results of HMMER server.The results of RDP analysis showed that there were no reorganization events in OSPVs.

While the ITRs of the OsPVs showed significant differences from those of the GPVs, MDPV, and NGPV, the biological significance of this remains unclear. The ITR not only functions as an origin of genome replication, but also contains several transcription factor binding sites, including E-box, ATF (activating transcription factor)/CREB (cyclic AMP-responsive element binding), and MLTF (upstream stimulate factor) [6,11]. Whether the nucleotide differences in the ITRs of OsPV have an effect on the transcription efficiency or virulence remain to be investigated. Numerous short repeat motifs and transcription factor binding sites are distributed in the ITRs of MDPV and GPV [6]. The OsPV-YN and OsPV-BJ strains have 15nt deletion (CTTCCGGTCATGTGA) at the left ITR, the distance between the repeat motif TTCCGGT and the transcription factor binding site E-box has been changed [12].

Some parvoviruses elicit DNA damage response (DDR) pathways, activation of DDR pathways is triggered by the complex hairpin structures in the genome [13]. The interplay of the virus replication cycle and the DDR machinery may play a critical role in viral pathogenesis [13].

The left ITR contains the replication starting point of viral DNA, the terminal resolution site (trs), and many repetitive sequences and domains consistent with the recognition motif of transcription factors [6,14]. The OsPV-YN, OsPV-BJ, and OsPV-SJZ strains have 15nt deletion (5’-GTCACGTGACAGGAA-3’) at 279nt and E-box motifs (CACATG) are widely involved in gene transcriptional regulation; thus, affecting cell cycle, immune response, and other physiological and pathological processes [15].

According to previous reports, GPV infects only geese and Muscovy ducks, but parvoviruses have a wide host range [4,16,17,18]. Avian parvoviruses have been detected in different areas of China, but this is the first report of a parvovirus in ostriches. However, whether OsPV is the pathogen of ostrich paralysis remains uncertain.

## Figures and Tables

**Table 1:** Detection results of ostrich parvovirus (OsPV) in clinical samples.

Place of Origin	Date	Number of Samples	Positive Rate	Total of Samples per City	Total Positive Rate
Beijing	2018.6	4	4/4	25	25/25
	2019.6	15	15/15		
	2019.7	6	6/6		
Yunnan	2018.6	2	2/2	2	2/2
Hebei	2018.7	2	2/2	30	29/30
	2018.8	12	12/12		
	2018.8	9	9/9		
	2019.7	6	6/6		
	2019.8	1	0/1		
Shanxi	2018.8	11	11/11	11	11/11

**Table 2:** Gene homology comparisons with other avian parvoviruses.

	MDPV ^a^ (FM Strain)	GPV ^b^ (SYG61v Strain)	NGPV ^c^ (SDLY1602 Strain)	GPV ^d^ (B Strain)	GPV ^e^ (RC16 Strain)	NGPV ^f^ (HuN18 Strain)	MDPV ^g^ (JH10 Strain)
whole genome	80.8–81.3%	94.1–94.4%	94.4–94.7%	97.7–97.9%	99.2–99.6%	95.5–95.9%	86.0–86.3%
Rep sequence	82.3–82.9%	93.7–94.4%	95.9–96.4%	98.6–99.3%	99.0–99.9%	95.9–96.3%	82.6–83.2%
Cap sequence	80.3–80.4%	95.3–95.5%	94.5–94.8%	96.2–96.6%	99.3–99.6%	96.0–96.3%	89.6–89.8%

a. The MDPV FM strain of Muscovy duck parvovirus (MDPV), discovered in Hungary in 1993 by Zadori et al. [6]; b. SYG61v strain (vaccine strain) of goose parvovirus (GPV); c. novel goose parvovirus-related virus (NGPV) strain SDLY1602 responsible for duck tongue disease, found in Shandong province in 2016 by Li et al. [7]; d. GPV strain B isolated from grey goose in Hungary [6]; e. GPV-RC16 strain from Chongqing reported in 2017 by Liu et al. [1,8]; f. the NGPV HuN18 strain of Novel goose parvovirus (NGPV), discovered in Hunan province in 2018 by Wan C et al. [9]; g. MDPV strain JH10 isolated from Muscovy duck in China [10].

## Supplementary Materials

The following are available online at https://www.mdpi.com/1999-4915/12/3/334/s1, Table S1: GPV and MDPV isolates used in this study for sequence comparison.
