# Supplementary material for: Whole Genome Characterization and Genetic Evolution Analysis of a New Ostrich Parvovirus
Source: Viruses. 2020 Mar 19;12(3):334. doi: 10.3390/v12030334 (PMC7150892; doi:10.3390/v12030334)
Supplement: Supplementary file 1 [file viruses-12-00334-s001.pdf]

Whole Genome Characterization and Genetic Evolution Analysis of a New Ostrich Parvovirus

Kunpeng Yuan [1], Dongdong Wang [2], Qingdong Luan [1], Ju Sun [2], Qianwen Gao [1], Zhiyao Jiang [1], Shouchun Wang [1], Yijun Han [1], Xueting Qu [1], Yueying Cui [1], Shimei Qiu [1], Youxia Di [3], Xiaoyi Wang [3], Shige Song [3], Peiheng Wang [3], Shilong Xia [3], Yongle Yu [4], Weiquan Liu [4,*] and Yanbo Yin [1,*]

[1] College of Veterinary Medicine, Qingdao Agricultural University, Qingdao 266019, China; 18306390700@163.com (K.Y.); lqddmj@126.com (Q.L.); gqwdmj2020@163.com (Q.G.); jzydmj@sina.com (Z.J.); wangshouchun2011@sina.com (S.W.); hyjdmj@souhu.com (Y.H.); qxtdmj@aliyun.com (X.Q.); cyydmj@tom.com (Y.C.); qsmdmj@tom.com (S.Q.);

[2] Qingdao Bolong Experimental Animal Co., Ltd., Qingdao 266225, China; wdddmj@126.com (D.W.); sjdmja@126.com (J.S.)

[3] China Ostrich Farming and Development Association, Beijing 100026, China; diyouxiayouxiang@163.com (Y.D.); wangxiaoyiyx2020@163.com (X.W.); ssgyouxiang2020@163.com (S.S.); wph2020@163.com (P.W.); xslyx2020@163.com (S.X.)

[4] College of Biological Sciences, China Agricultural University, Beijing 100193, China; yylyx2020@163.com (Y.Y.)

* Correspondence: weiquan8@126.com (W.L.); yanoyin2011@163.com (Y.Y.)

Supplementary Table 1. GPV and MDPV isolates used in this study for sequence comparison.

Virus	Strain Name	Host	Place of Origin	GenBank Accession No.
Classical GPV	B	Anser anser	Hungary, 1995	U25749
Classical GPV	E	Goose	Anhui, China, 2012	KC184133
Classical GPV	GDaGPV	Goose	Guangdong, China, 1978	HQ891825
Classical GPV	SH	Anser anser	Shanghai, China, 2009	JF333590
Classical GPV	SYG61v	Goose	China,1961	KC996729
Classical GPV	VG32/1	Goose	Germany	EU583392
Classical GPV	Y	Muscovy duck	Anhui, China, 2011	KC178571
Classical GPV	YZ99-6	Goose	Jiangsu, China, 1999	KC996730
Classical GPV	LH	Goose	China, 2012	KM272560
Classical GPV	SHFX1201	Swan	Shanghai, China 2012	KC478066
Classical GPV	06-0329	Goose	Taiwan, 2006	EU583391
Classical GPV	82-0321v	Goose	Taiwan, 1982	EU583389

Classical GPV	RC16	Goose	Chongqing, China 2016	KY475562
Classical GPV	WX	Goose	Anhui, China 2013	KR091959.1
Classical GPV	YZ	Goose	Anhui, China 2013	KR091960.1
Classical GPV	Yan-2	yan goose	Anhui, China ,2013	KR136258.1
Classical GPV	M15	Mule duck	China,2015	KU844283
Novel GPV	SDLY1512	Cherry Valley ducks	Linyi, Shandong, China, Dec. 2015	MF441221.1
Novel GPV	SDLY1602	Cherry Valley ducks	Linyi, Shandong, China, Feb. 2016	MF441222.1
Novel GPV	SDHZ1604	Cherry Valley ducks	Heze, Shandong, China, Apr. 2016	MF441223.1
Novel GPV	SDDY1605	Cherry Valley ducks	Dongying, Shandong, China, May. 2016	MF441224.1
Novel GPV	AH1606	Cherry Valley ducks	Haozhou, Anhui, China,June. 2016	MF441225.1
Novel GPV	JS1603	Cherry Valley ducks	Peixian, Jiangsu, China, Mar. 2016	MF441226.1
Novel GPV	AH1605	Cherry Valley ducks	Suzhou, Anhui, China, May. 2016	MF441227.1
Novel GPV	HuN18	Linwu Sheldrake	Linwu, Hunan, China, 2018	MK736656
Classical MDPV	P	Muscovy duck	Fujian China, 1988	KU844281
Classical MDPV	P1	Muscovy duck	China, 2015	KU844282
Classical MDPV	GX5	Muscovy duck	Guangxi, China, 2011	KM093740
Classical MDPV	FM	Cairina moschata	Hungary 1993	NC006147
Classical MDPV	FZ91-30	Muscovy duck	China, 1991	KT865605
Classical MDPV	SAAS-SHNH	Muscovy duck	Shanghai, China, 2012	KC171936
Classical MDPV	JH10	Muscovy duck	Zhejiang, China, 2010	MH807698
Classical MDPV	JH06	Muscovy duck	Zhejiang, China, 2006	MH807697
Classical MDPV	PT97	Muscovy duck	Fujian, China, 1997	KY511293
Classical MDPV	ZW	Muscovy duck	Zhejiang, China, 2006	KY744743
